# Prevalence and correlates of gambling problems among forensic psychiatric patients: evidence of elevated rates from a multi-site study

**DOI:** 10.3389/fpsyt.2026.1931287

**Published:** 2026-10-20

**Authors:** Jakob W. Eriksen, Lisbeth U. Sørensen, Søren Kristiansen, Morten D. Terkildsen, Rasmus M. Hansen, Thomas Marcussen, Mette Brandt-Christensen, Asbjørn Storgaard, Kaare B. Wellnitz, Lisbeth Frostholm

**Affiliations:** 1 Research Clinic on Gambling Disorders, Aarhus University Hospital, Aarhus, Denmark; 2 Department of Forensic Psychiatry, Aarhus University Hospital, Aarhus, Denmark; 3 Department of Clinical Medicine, Health, Aarhus University, Aarhus, Denmark; 4 Department of Sociology and Social Work, Aalborg University, Aalborg, Denmark; 5 DEFACTUM - Social & Health Services and Labour Market, Central Denmark Region, Aarhus, Denmark; 6 Competence Centre for Forensic Psychiatry, Copenhagen University Hospital, Capital Region of Denmark, Roskilde, Denmark; 7 Department of Law, University of Southern Denmark, Odense, Denmark; 8 Department of Functional Disorders, Aarhus University Hospital, Aarhus, Denmark

**Keywords:** behavioral addiction, forensic psychiatry, gambling, gambling disorder, prevalence, problem gambling, severe mental illness

## Abstract

**Introduction:**

Gambling problems (GP) remain underexplored among forensic psychiatric patients (FPPs). Given the associations between GP, mental distress, and criminal behavior, it is imperative to investigate GP in this population.

**Aims:**

This study aimed to estimate the prevalence of gambling and GP among FPPs in Denmark and explore associations between GP and sociodemographic, behavioral, and clinical variables.

**Methods:**

A cross-sectional survey was conducted, including 214 FPPs across 19 hospital units in two major Danish Health Regions. The primary measures were past-year gambling and GP assessed via the Problem Gambling Severity Index (PGSI). Secondary measures included gambling behavior (e.g., expenditure, frequency, self-exclusion), sociodemographic (e.g., age, gender, educational attainment), financial (e.g., consumer loans, monetary savings), and clinical variables (e.g., length of stay, psychiatric diagnoses). Gambling FPPs were asked if they had felt compelled to commit crime due to gambling during the past 12 months. Correlates of GP were identified by comparing higher-risk (PGSI ≥ 3) to lower-risk (PGSI ≤2) gambling FPPs. Prevalence was compared to a gender- and age-matched normative sample from a recent Danish national gambling survey.

**Results:**

The prevalence of past-year gambling among FPPs was 46% (95% CI = 39-53%), while the prevalence of severe GP (PGSI ≥ 8, indicative of gambling disorder) was 12% (95% CI: 8.1-17%). Relative to lower-risk gambling FPPs (PGSI ≤2), higher-risk FPPs (PGSI ≥ 3) were more likely to be male, to express poorer financial conditions, and to have felt compelled to engage in criminal behavior due to gambling. GP prevalence was markedly higher among FPPs than in the normative sample, where 1.8% reported severe GP (PGSI ≥ 8) (95% CI: 1.3-2.5%).

**Discussion and Conclusion:**

To our knowledge, this is the first study to examine the prevalence of gambling and GP among FPPs, and the rates were considerable. Given the consequences that GP may have on FPP health and economy, and thus recovery, our findings underscore the potential benefits of screening for and addressing GP in forensic psychiatric care. There is a strong need for further research to inform tailored interventions, improve treatment, and mitigate the potential impact of gambling on recidivism risk.

## 1 Introduction

Forensic psychiatry offers care to foster recovery, reduce recidivism, and support rehabilitation for individuals with severe mental illness who have committed serious criminal offenses (1). A majority of forensic psychiatric patients (FPPs) have limited socioeconomic resources, low educational attainment, and are diagnosed with a psychotic disorder. A substantial proportion suffer from comorbid substance use disorder (2–5). An unexplored clinical domain, however, is that of behavioral addictions. Considering the well-documented connections between behavioral addictions and poor mental, social, and physical health (6, 7), this gap in current research is notable.

The first behavioral addiction to attain official diagnostic status is gambling disorder, characterized by recurrent maladaptive patterns of gambling and related harmful behaviors that persist despite significant negative consequences, as defined in the Diagnostic and Statistical Manual of mental disorders 5th edition (DSM-5) (8). Gambling disorder represents the severe end of a continuum of harmful gambling behavior, the consequences of which are commonly referred to as gambling problems (GP) (9, 10). In this paper, GP denotes any level of gambling-related harm, whereas problem gambling denotes levels of harm indicative of gambling disorder.

The absence of initiatives to systematically map GP in FPP populations suggests that GP receive minimal attention. This notion is further corroborated by qualitative interviews conducted with forensic psychiatric staff and patients, presented in a separate paper as part of the present research project (11). Insofar as gambling is accessible to FPPs across countries and jurisdictions, an absence of clinical and scientific attention is concerning. In Denmark, since the liberalization of the gambling market in 2012 (12), online gambling has been legally available with a range of national and international operators offering gambling products to Danish citizens. This is compounded by a recent growth in online offshore gambling (13). Patients admitted to psychiatric wards in Denmark have full access to the internet. This also applies to FPPs, including those placed in medium-secure units, who therefore have ample opportunity to engage in online gambling.

GP are consistently associated with male gender, young to middle age, singlehood, unemployment, and low educational attainment (14, 15), closely mirroring the general profile of FPPs (5, 16). Furthermore, GP are highly comorbid with psychiatric disorders that are prevalent among FPPs (17, 18). A Danish study (19) found considerable prevalence rates of schizophrenia spectrum disorders (10.2%), personality disorders (10.6%), and substance use disorders (13.0%) among individuals seeking treatment for GP. Moreover, GP are closely linked to criminal behavior. A recent meta-analysis (20) found a 30.8% prevalence of GP among imprisoned individuals across 26 studies. This is markedly higher than prevalence rates in general populations (2.4-8.7%) (21, 22) and raises concerns that severely mentally ill individuals receiving court-mandated treatment could also be at elevated risk of GP.

Irrespective of FPP susceptibility, the associated harms of GP alone warrant systematic investigation in this population. GP are linked to distress and psychosocial maladjustment (7, 23), potentially obstructing mental health progression. Moreover, GP compromise financial stability, a key factor in recovery and rehabilitation (24). Finally, GP have been shown to predict subsequent criminal activities, including theft, fraud, and burglary (25, 26) and to be associated with increased recidivism risk among ex-offenders (27). Given these links, assessing GP prevalence within forensic psychiatric populations is essential for understanding its position in recovery and provides a foundation for developing targeted prevention and rehabilitation strategies tailored to the specific needs of this high-risk group.

The aims of the present study were to 1) estimate the prevalence of gambling and GP among FPPs in Denmark and 2) explore associations between GP and FPP sociodemographic, behavioral, and clinical variables.

## 2 Materials and methods

This project is preregistered via Open Science Framework (OSF) DOI: https://doi.org/10.17605/OSF.IO/6YF5S.

### 2.1 Study design

We conducted a cross-sectional survey. Data were collected from June 1, 2024, to January 15, 2025, at multiple sites throughout Central and Capital Denmark Regions, the two largest of five Danish Health Regions.

### 2.2 Participants

The target population comprised all FPPs in the Central Denmark Region (N = 910 according to administrative trajectory markers for individuals under forensic psychiatric measures in regional registers of partial completeness (28)), as well as all FPPs in medium secure wards in the Capital Denmark Region (n = 156) between June 1, 2024, and January 15, 2025. The study aimed at recruiting the largest possible number of eligible FPPs throughout the data collection period.

#### 2.2.1 Eligibility criteria

Participants had to be a) FPPs, b) at least 18 years of age, c) able to speak Danish or Greenlandic, d) registered in Central Denmark Region care or at medium secure forensic psychiatric wards in Capital Denmark Region, e) able to voluntarily consent to participating in the study. Patients were excluded if they a) were unable to participate due to intake of psychoactive substances, b) suffered brain damage, neurodegenerative disorders, or cognitive impairments compromising their ability to complete the questionnaire, or c) were considered too ill to participate in the study at the discretion of the chief physician or primary nurse (e.g., distressed, delusional, somatically ill).

### 2.3 Procedures

Recruitment and data collection were organized in collaboration with management and staff at six hospital sites, mutually consisting of 19 units, each with an appointed “unit coordinator” and a group of data collectors among staff assisting the first author (JWE) in completing data collection. All data collectors underwent 75 minutes of training lectured by JWE. FPPs received verbal and written information about the study and consented voluntarily for participation and for the retrieval of information from electronic patient records. Questionnaires were completed at hospital sites via tablets. When necessary, data collectors assisted participants in reading questions and marking responses. Participants were offered a snack and a soft drink upon participation, and those completing the Problem Gambling Severity Index (PGSI) (29) were given the choice of receiving a one-page handout with personalized normative feedback with brief advice on how to reduce gambling behavior.

### 2.4 Data sources

Data were collected via a self-report questionnaire and participants’ electronic patient records. A normative gender- and age-matched sample for comparison was drawn randomly from a national survey of GP in Denmark (30).

### 2.5 Measures

#### 2.5.1 Gambling behavior

Inspired by The Banff, Alberta Consensus (31), we included past-year gambling (yes/no) as the primary measure of gambling behavior, and gambling frequency (monthly), monetary expenditure (monthly), and time expenditure (weekly) as secondary measures. Gambling modality (digital/analog) and type (e.g., casino, sports betting, slot machine, poker) were also surveyed.

#### 2.5.2 The problem gambling severity index

In accordance with guidelines (32), PGSI was chosen as the primary measure of GP (29). PGSI consists of nine items rated on a 4-point Likert scale (never, sometimes, most of the time, almost always) resulting in sum scores between 0 and 27, and identifies four categories with reference to GP: non-problem (0), low risk (1-2), moderate risk (3-7), and problem gambling (≥8), the latter indicative of gambling disorder. PGSI is widely employed in surveys of GP (22).

#### 2.5.3 Additional indicators of gambling harm

Participants were asked if they had ever experienced GP before entering forensic psychiatric services (yes/no), whether they had ever sought help for GP, and whether they were currently using national gambling self-exclusion services. Additionally, participants were asked whether they had felt compelled to engage in criminal behavior due to gambling within the past year.

#### 2.5.4 Sociodemographic and behavioral variables

Several sociodemographic and behavioral variables were collected via self-report. This included age, gender, country of birth, marital status, housing, educational attainment, level of income, monthly monetary savings, and consumer loans.

#### 2.5.5 Clinical variables

Hospitalization status (inpatient/outpatient) was recorded via the questionnaire, whereas psychiatric diagnoses and length-of-stay (days from index verdict to study participation) were retrieved from the electronic patient records. Psychiatric diagnoses were grouped according to the International Classification of Diseases (ICD-10) (33).

#### 2.5.6 General population data

Upon requesting the Danish Gambling Authority, we received original data from their recent national GP survey using PGSI (30). Of 10,000 randomly selected adults, 3,861 participated. This enabled the construction of an age- and gender-matched normative sample for comparison with our FPP sample on past-year gambling (yes/no) and PGSI ratings.

### 2.6 Statistical analysis

Descriptive statistics included means and standard deviations (SD) for continuous variables and counts and percentages (%) for categorical variables.

Prevalence of gambling among FPPs was estimated as proportion having gambled in the previous year, while prevalence of GP was estimated as proportion indexed with low risk, moderate risk, and problem gambling according to PGSI, all with 95% exact binomial confidence intervals.

To compare the prevalence of past-year gambling and GP among FPPs and the general population, a matched sample from the general population sample was made. Specifically, for each FPP with complete data on gender and age (n=209), ten individuals from the general population sample were matched on gender (male/female) and age-group (of ten-year age span: 20-29, 30-39,…70-79). For different FPPs, the same matched individual could be sampled, but for individual FPPs, ten unique matches were made. Ultimately, a matched sample of 2,090 individuals was created, and prevalence rates of past-year gambling among FPPs and the matched sample were compared using a Wald two-sample proportion test, whereas differences in the distribution of PGSI risk levels between FPPs and the matched sample were evaluated using chi-square.

Lastly, associations between GP and sample characteristics were evaluated by dividing past-year gambling FPPs into lower-risk and higher-risk gamblers according to PGSI (PGSI ≤ 2 vs. PGSI ≥ 3). Higher-risk and lower-risk gamblers were then compared on gambling characteristics as well as clinical, financial, and sociodemographic characteristics. Pearson chi-square or Fisher’s exact tests were employed for categorical variables, whereas independent t-tests were employed for continuous variables. To aid in the interpretation of direction and magnitude of association, counts (%) or means (SD) were reported for each combination of risk group and respective sample characteristic. For each comparison, mean differences (MD) were reported for continuous characteristics, odds ratios were reported for binary characteristics, and chi-square values were reported for multinomial characteristics.

All statistical analyses were done in Stata version 19.5.

### 2.7 Ethics

The study was conducted in accordance with the Declaration of Helsinki (34). The Central Denmark Region Ethics Committee determined that the study was exempt from formal approval under Danish Law (case no. 1-10-72-6-24). The study was submitted to the internal register (case no. 1-16-02-87-25) in accordance with the regional protocol for GDPR compliance. All subjects were informed about the study and gave their informed consent. Due to GDPR, no data were available for non-responders in the present study.

## 3 Results

### 3.1 Recruitment

Across 19 contributing units at six hospitals, 641 possible participants were identified, of whom 486 were invited, and 214 participated, corresponding to 44% of those invited (flow diagram, **Figure 1**). In all, 179 participants consented to the retrieval of data from electronic patient records. A total of 134 of 214 participants (63%) completed the questionnaire independently, whereas 80 (37%) did so with some assistance.

**Figure 1:**
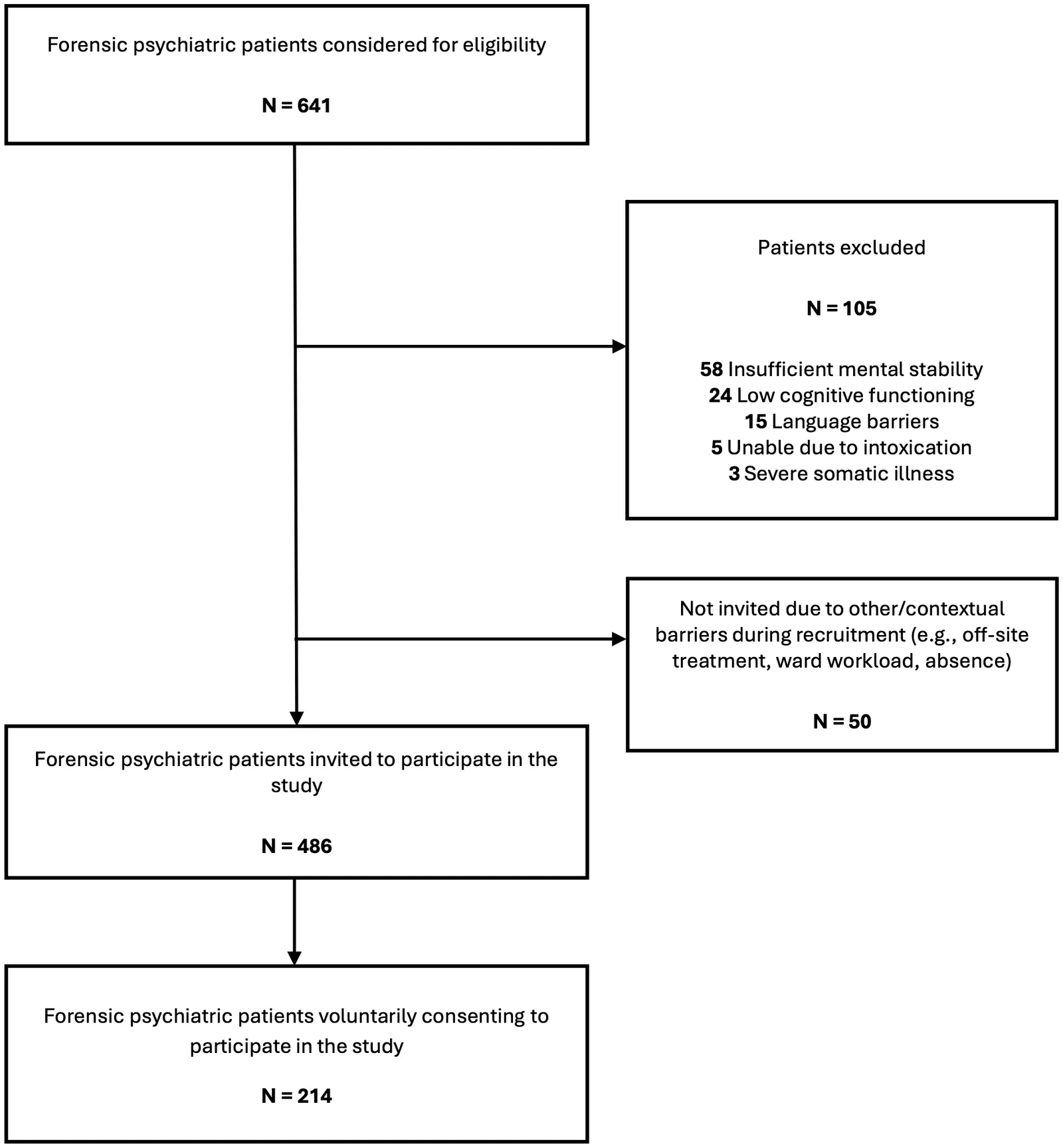
Flow of participant recruitment from June 1, 2024, to January 15, 2025.

### 3.2 Study sample characteristics

The mean age was 38.2 years (SD = 11.5), 20% (n = 43) were female, and 77% (n = 164) were born in Denmark (**Table 1**). A total of 41% (n = 87) were inpatients, and among consenters for the retrieval of patient record data on length-of-stay (n = 136), 52% (n = 71) received their index verdict at least 3 years ago. Among consenters for the retrieval of data on diagnoses (n = 177), 65% had a psychotic disorder (F2, ICD-10) as their primary diagnosis, and 40% were diagnosed with substance use disorders (F1, ICD-10) either as primary or secondary diagnosis. Less than 3 were diagnosed with gambling disorder (F63.0, ICD-10).

**Table 1:** Sample characteristics.

N	Mean (*SD*) n (%)	n missing
	214	
Sociodemographics		
Age, years	38.21 (11.50)	4 (1.9%)
Female^a^	43 (20%)	1 (0.5%)
Country of birth: Denmark	164 (77%)	1 (0.5%)
Marital status: In relationship	23 (11%)	13 (6.1%)
Educational attainment > Primary school	79 (37%)	1 (0.5%)
Monthly income > 10,000 DKK	154 (72%)	5 (2.3%)
Clinical characteristics		
Hospitalization status: Inpatient	87 (41%)	0 (0.0%)
Length-of-stay		78 (36%)
< 1 year	23 (11%)	
1–2 years	16 (7.5%)	
2–3 years	26 (12%)	
3–4 years	13 (6.1%)	
4–5 years	15 (7.0%)	
5+ years^b^	43 (20%)	
Primary ICD-10 diagnosis		44 (21%)
Organic psychiatric disorders, F0	7 (3.3%)	
Substance use disorders, F1	9 (4.2%)	
Psychotic disorders, F2	115 (54%)	
Affective disorders, F3	10 (4.7%)	
Neurotic disorders, F4	≤3 (≤1.4%)	
Personality disorders, F6	7 (3.3%)	
Developmental disorders, F8	7 (3.3%)	
ADHD and conduct disorders, F9	≤3 (≤1.4%)	
SUD diagnosis^c^	70 (33%)	44 (21%)
Gambling disorder diagnosis^c^*	≤3 (≤1.4%)	44 (21%)
Gambling characteristics		
Past year gambling	98 (46%)	0 (0.0%)
PGSI risk level		1 (0.5%)
Non-problem gambler (0)	32 (15%)	
Low risk gambler (1-2)	18 (8.4%)	
Moderate risk gambler (3-7)	21 (9.8%)	
Problem gambling (≥8)	26 (12%)	
Pre-admission gambling problems	34 (16%)	7 (3.2%)
Gambling help-seeking ever^d^	9 (4.2%)^b^	119 (56%)
Consulted forensic psychiatry for help^e^*	≤3 (≤1.4%)	205 (96%)
Current use of national gambling exclusion^f^	27 (13%)	106 (50%)
Past-year criminal intent due to gambling^d^	10 (4.7%)	117 (55%)

ADHD, Attention deficit hyperactivity disorder; DKK, Danish Currency (Danske Kroner); N, number of participants; PGSI, the Problem Gambling Severity Index; SD, standard deviation; SUD, substance use disorder. ^a^Participants were presented with a category of "other gender", but none chose this option. ^b^Due to a reorganization of regional databases in February 2019, cases with a duration exceeding five years could not be dated. ^c^Counting both primary and secondary diagnoses, ^d^Only FPPs reporting past-year gambling were asked this, ^e^Only FPPs reporting help-seeking were asked this. ^f^FPPs, having never gambled, were not asked this. *Cells with counts ≤3 have been suppressed.

### 3.3 Gambling and gambling problem prevalence rates

The prevalence of past year gambling was 46% (95% CI = 39-53%), whereas the prevalence of GP (i.e., PGSI score > 0) was 30% (95% CI = 24-37%), with 8% reporting low risk gambling (95% CI = 5-13%), 10% reporting moderate risk gambling (95% CI = 6-14%), and a total of 12% showing PGSI sum-scores equivalent to problem gambling (95% CI = 8-17%) indicative of gambling disorder. Overall, 16% (95% CI = 11-21%) of the sample reported having experienced some GP before forensic psychiatric admission, and 13% (95% CI = 9-18%) of the sample were currently using national gambling self-exclusion services (**Table 1**).

### 3.4 Gambling FPPs and variables associated with GP

The present subsection and **Table 2** outline our findings on FPPs reporting past-year gambling (n = 98). For comparisons of gambling and non-gambling FPPs, see **Supplementary Table 1**.

**Table 2:** Comparison of higher-risk vs. lower-risk gambling forensic psychiatric patients.

	All gambling FPPs	Lower-risk^a^ gambling FPPs	Higher-risk^b^ gambling FPPs	Effect size^c^ OR / *MD* [95%CI]	p-value
N	97^d^	50	47		
Mean (SD) / n (%)					
*Subgroup comparisons*					
Gambling characteristics					
PGSI score	4.8 (5.8)	0.4 (0.6)	9.4 (5.1)	–	–
Past month gambling frequency					
No gambling	28 (29%)	14 (28%)	14 (30%)	χ^2^(3) = 2.0^j^	0.578
Once	22 (23%)	14 (28%)	8 (17%)		
A couple of times	21 (22%)	9 (18%)	12 (26%)		
A couple of times a week or more	28 (29%)	13 (26%)	13 (28%)		
Past-month gambling expenditure > 1,000 DKK	14 (20%)	0 (0.0%)	14 (42%)	–	**<0.001**
Past week gambling volume > 1 hour	23 (35%)	7 (20%)	16 (52%)	4.3 [1.4, 12.7]	**0.007**
Gambling modality				χ^2^(2) = 7.0^j^	**0.031**
Online only	56 (61%)	25 (53%)	31 (69%)		
Offline only	21 (23%)	16 (34%)	5 (11%)		
Online and offline	15 (16%)	6 (13%)	9 (20%)		
Most frequent gambling type (online)^e^				χ^2^(6) = 15.5^j^	**0.016**
Slot machines	21 (30%)	7 (23%)	14 (35%)		
Casino games*	14 (20%)	≤3 (≤9.8%)	11–14 (28–30%)		
Sports betting	18 (25%)	11 (36%)	7 (18%)		
Poker*	7 (9.9%)	≤3 (≤9.8%)	4–7 (10–18%)		
Lotteries or scratch tickets*	9 (13%)	6–9 (19–29%)	≤3 (≤7.5%)		
Skin gambling*	≤3 (≤4.2%)	≤3 (≤9.8%)	0 (0.0%)		
Other*	≤3 (≤4.2%)	≤3 (≤9.8%)	0 (0.0%)		
Current use of national gambling exclusion	21 (30%)	4 (12%)	17 (47%)	6.5 [1.9, 22.3]	**0.002**
Pre-admission gambling problems	26 (30%)	5 (11%)	21 (53%)	9.3 [3.0, 28.3]	**<0.001**
Gambling help-seeking ever*	9 (9.5%)	≤3 (≤6.0%)	6–9 (13–20%)	10.1 [1.2, 84.4]	**0.011**
Past-year criminal intent due to gambling	10 (10%)	0 (0.0%)	10 (21%)	–	**<0.001**
Financial characteristics					
Monthly income > 10,000 DKK	74 (78%)	39 (80%)	35 (76%)	0.8 [0.3, 2.2]	0.681
Monthly monetary savings (yes)	52 (59%)	35 (76%)	17 (41%)	0.2 [0.1, 0.5]	**<0.001**
Consumer loans currently (yes)	27 (30%)	10 (21%)	17 (42%)	2.7 [1.1, 6.8]	**0.035**
Sociodemographics					
Age^f^	35.8 (10.2)	35.9 (10.8)	35.6 (9.6)	*0.3 [−3.9, 4.5]*	0.887
Female^g^	17 (18%)	13 (26%)	4 (8.7%)	3.7 [1.1, 12.3]	**0.027**
Country of birth: Denmark	76 (78%)	41 (82%)	35 (75%)	0.6 [0.2, 1.7]	0.368
Marital status: In relationship	9 (10%)	5 (11%)	4 (8.9%)	0.8 [0.2, 3.0]	0.699
Educational attainment > Primary School	30 (31%)	20 (40%)	10 (22%)	0.4 [0.2, 1.0]	0.054
Clinical characteristics					
Length-of-stay				χ^2^(5) = 7.9^j^	0.165
< 1 year	13 (20%)	7 (21%)	6 (19%)		
1–2 years	10 (16%)	4 (12%)	6 (19%)		
2–3 years	13 (20%)	6 (18%)	7 (23%)		
3–4 years*	6 (9.4%)	3–6 (9–18%)	≤3 (9.7%)		
4–5 years*	7 (11%)	≤3 (≤9%)	4–7 (13–23%)		
5+ Years^h^	15 (23%)	8 (24%)	7 (23%)		
Hospitalization status: Inpatient	43 (44%)	25 (50%)	18 (38%)	0.6 [0.3, 1.4]	0.246
SUD diagnosis^i^	35 (43%)	14 (34%)	21 (51%)	2.0 [0.8, 4.9]	0.118

CI, confidence interval; DKK, Danish Currency (Danske Kroner); N, number of participants; MD, mean difference; OR, odds ratio; PGSI, the Problem Gambling Severity Index; SD, standard deviation; SUD, substance use disorders. ^a^Gambling FPPs with PGSI scores equal to or less than 2, ^b^Gambling FPPs with PGSI scores equal to or higher than 3. ^c^Continuous variables were analyzed using independent t-tests and are presented as MD. Categorical variables were analyzed using chi-square tests or Fisher's exact tests, as appropriate. For binary variables, ORs are presented, whereas for multinomial variables, the chi-square (χ²) test statistic is presented, as ORs are not estimated for these variables. For binary variables with zero counts in one or more cells, odds ratios could not be calculated. All tests were based on FPPS responding to the given set of questions and thus did not include differences in propensity to be missing. ^d^One of 98 FPPs excluded from analyses due to missing PGSI data. ^e^Owing to very small cell sizes, offline type is not reported. ^f^Data on age were missing for two individuals in the lower-risk group, resulting in an analytic n of 95. ^g^Participants were presented with the option of "other gender", but none chose this. ^h^Due to a reorganization of regional databases in February 2019, cases with a duration exceeding five years could not be dated. ^i^Counting both primary and secondary diagnoses. ^j^The chi-square test statistic is presented, as ORs are not calculated for multinomial variables. *Cells with counts ≤3 have been suppressed. Additional cells have been suppressed to prevent back-calculation. p-values < 0.05 are displayed in bold.

Overall, 66% of the gambling subset of FPPs reported some GP (95% CI = 56-76%) and 27% had PGSI sum-scores equivalent to problem gambling (95% CI = 18-36%). Online slot machines were the most frequent type of gambling. Relative to lower-risk gamblers, higher-risk gamblers were more likely to be male (91% vs. 74%, *p* = 0.027), were more likely to having felt compelled to engage in criminal activity within the past year due to gambling (21% vs 0%, *p* < 0.001), to have a history of help-seeking for GP (17% vs. 2.0%, *p* = 0.011), and to currently use gambling self-exclusion services (47% vs. 12%, *p* = 0.002). No difference was found in level of income (*p* = 0.681), but having consumer loans was twice as common among higher-risk gamblers (42% vs. 21%, *p* = 0.035), and the propensity to set aside money monthly was significantly lower among higher-risk gamblers (41% vs. 76%, *p* < 0.001). No individuals in the lower-risk group spent over DKK 1,000 (≈ EUR 184) monthly on gambling, whereas 42% did so in the higher-risk group (*p* < 0.001).

### 3.5 Gambling among FPPs and in the general population

Compared to the gender- and age-matched normative sample, past-year gambling was less common among FPPs (46% vs. 54%, *p* = 0.016), however relative to the normative sample, the prevalence of GP (PGSI > 0) was observed to be nearly two-fold among FPPs (30% vs. 18%), whereas the rate of problem gambling (PGSI ≥ 8) was more than six-fold (12% vs.1.8%). Estimates, confidence intervals, and comparisons are displayed in **Table 3**. The overall distribution of past-year PGSI risk levels was significantly different between the two populations (χ2(4) = 107.6, *p* < 0.001).

**Table 3:** Comparison of forensic psychiatric patients and a gender- and age-matched general population sample.

	Forensic psychiatric patients		Matched sample		Effect size^a^ [95%CI]	p-value
N	209^b^		2090^c^			
% (n) [95% CI]						
Past year gambling	45% (95)	[39-52%]	54% (1,133)	[52-56%]	9% [2-16%]	**0.016**
PGSI risk level					χ^2^(4) = 107.6	**<0.001**
Non-problem gambling	15% (31)	[10-20%]	37% (764)	[35-39%]		
Low risk gambling	8.1% (17)	[4.8-13%]	10% (213)	[8.9-12%]		
Moderate risk gambling	10% (21)	[6.3-15%]	5.7% (118)	[4.7-6.7%]		
Problem gambling	12% (25)	[7.9-18%]	1.8% (38)	[1.3-2.5%]		
Non-gamblingd	55% (115)	[48-62%]	46% (957)	[44-48%]		

N, number of participants; PGSI, the Problem Gambling Severity Index. ^a^For past year gambling presented as difference in proportions according to Wald test. For PGSI risk levels displayed as chi-square (χ²) value. ^b^Five FPPs missing data on either gender or age were excluded from analyses. ^c^Matched 1:10 with equal gender distribution and an age margin of +/- 5 years for each matched individual (further details in analysis section) ^d^Including one gambling FPP missing PGSI data. p-values < 0.05 are displayed in bold.

### 3.6 Additional analyses

We examined whether there were differences between higher-risk and lower-risk gambling FPPs in their propensity to request personalized normative feedback. Overall, 51% (n = 49) of gambling FPPs requested personalized normative feedback, and higher-risk gamblers were more likely to do so than were lower-risk gamblers (67% vs. 36%, OR = 3.7, 95% CI [1.6, 8.6], *p* = 0.002).

Additionally, we examined whether primary gambling outcomes differed between independent and assisted questionnaire completers. Past-year gambling was more common among independent completers (54% vs. 31%, *p* = 0.001), however mean PGSI scores did not differ between independent and assisted completers (0.45 vs. 0.56, MD = -0.11, 95% CI [-0.34, 0.12], *p* = 0.579). Neither did the distribution of higher- and lower-risk gamblers (PGSI ≤ 2 vs. PGSI ≥ 3) (OR = 1.6, 95% CI [0.6, 3.9], *p* = 0.347).

## 4 Discussion

In response to calls for research on GP in vulnerable populations (35), this study investigated the prevalence and correlates of GP among FPPs in Denmark, addressing a largely overlooked issue in forensic psychiatry. With nearly half of FPPs reporting past-year gambling, gambling is common, albeit slightly less prevalent than in a gender- and-aged-matched normative sample. Despite this, GP were far more common among FPPs, and the proportion meeting criteria for problem gambling, indicative of gambling disorder, substantially exceeded that of the normative sample, indicating a particular vulnerability to the harmful consequences of gambling.

Apart from male gender, typical sociodemographic correlates of GP were not observed. This could be due to the similarities of the general FPP sociodemographic profile and GP risk factors, or it could be due to sample size. Of note, gamblers were younger than non-gamblers (see **Supplementary Table 1**), however risk level among gambling FPPs was not associated with age.

Comparing lower- and higher-risk gambling FPPs across central behavioral indicators provided important insights into how gambling may impede recovery. Higher-risk gamblers reported greater gambling expenditure and poorer financial conditions. Given the central role of financial security in both recovery and rehabilitation (24), this finding is particularly concerning. Compounding this, 21% of higher-risk gamblers, compared to none of the lower-risk gamblers, reported having felt compelled to commit crime as a consequence of gambling within the past year. While this finding should be interpreted cautiously, it suggests FPP gambling behavior as a potential contributor to recidivism risk, which warrants further investigation.

Use of the national gambling self-exclusion services was common among higher-risk gamblers, revealing how some FPPs act towards gambling-related harm. Our interviews with forensic psychiatric staff and patients suggest that, for the most part, FPPs do so autonomously (11). Contrasting this, less than 3 gambling FPPs had ever consulted forensic psychiatric staff for help, indicating barriers for the patients to utilize available professional resources. This is supported by our qualitative findings identifying barriers rooted both in patient perceptions, staff competencies, and structural factors (11). Future studies should examine these barriers more in depth and how they might be overcome. Our finding that a large proportion of higher-risk gambling FPPs in the present study requested personalized normative feedback suggests that, under certain conditions, they may be willing to receive feedback on their gambling habits.

This study appears to be the first mapping of GP among FPPs. Given the general sociodemographic profile of this population, heightened vulnerability is expected. Even though we examined associations with both hospitalization status and length of stay, it remains largely unclear to what extent institutional settings or structures in forensic psychiatric care might also contribute to the observed prevalence. While studies on GP among general psychiatric patients largely report rates exceeding those within general populations (36, 37), any impact of clinical or institutional settings is rarely assessed, and thus, there is limited evidence from which to draw parallels to the forensic psychiatric context on this question. A recent meta-analysis (38) concluded that hospitalization status (inpatient/outpatient) among individuals with psychotic disorders did not account for between-study heterogeneity in GP prevalence. Consistent with our finding among FPPs that GP did not differ by hospitalization status, this provides some suggestion that patient characteristics may be more influential than institutional settings per se.

Notably, across 12 studies Corbeil et al. (38) estimated an 8.7% prevalence of problem gambling among individuals with psychotic disorders (95% CI = 7.8%–9.7%). This estimate provides substantive context for interpreting the problem gambling prevalence rate among FPPs in our sample, where at least 54% of FPPs suffered a psychotic disorder as their primary diagnosis (65% of consenters to the retrieval of diagnostic data). Individuals with psychotic disorders appear to be particularly vulnerable to GP. Next to common sociodemographic risk factors, researchers propose that individuals with psychotic disorders may be prone to erroneous thinking or cognitive distortions revolving gambling, such as delusions of control or interpretive bias in explaining losses (39), which are known to correlate consistently with gambling problem severity (40). Also, a recent study based on polygenic risk scores suggests that genetic risk for schizophrenia is associated with increased risk of gambling disorder, pointing to a potential shared genetic liability between the two conditions (41). To contextualize our findings further, another recent meta-analysis (42) reported a pooled problem gambling prevalence estimate of 17% among individuals with substance use disorders, and in our sample, at least 33% were diagnosed with substance use disorders (40% of consenters to the retrieval of diagnostic data). Several dispositional factors have been identified as overlapping between GP and substance use problems, including difficulties with emotion regulation (43, 44), and reduced inhibitory control (45, 46). Taken together, prevalence rates and dispositional vulnerabilities documented in the broader gambling and addiction literature could contribute to the relatively high problem gambling prevalence of 12% observed in our sample, although this warrants further investigation.

Nevertheless, there may be important perspectives in examining the possible impact of structural and sociocultural aspects of forensic psychiatry on FPP gambling patterns. Treatment is compulsory, and admissions are generally of long duration, potentially allowing the settings to exert significant influence on behavior. In this regard, there are certain parallels to prison settings (e.g., prolonged confinement in highly restricted environments), where GP prevalence rates remain the highest observed (30%) (20). In our interview study (11), inpatient FPPs delineate how gambling serves social functions and is used both as stimulation and as a coping strategy in a routine-bound and restricted existence, providing evidence, at least from FPP perspectives, that social and structural factors shape gambling behavior. Similar accounts about social positioning and the abundance of unstructured time have been observed in populations of imprisoned individuals (47–49). Overall, beyond person-centered approaches, investigating the causal role of contextual factors in GP among forensic psychiatric patients, including the role of institutional environments, may help identify possible leverage points for intervention.

### 4.1 Implications

Given that one in four gambling FPPs met criteria indicative of gambling disorder and that gambling appears to draw some patients toward criminal behavior, the present study underscores the need for heightened awareness and clinical action addressing gambling among FPPs as both clinically and criminologically relevant behavior. Moreover, fewer than three FPPs had ever received a formal diagnosis of gambling disorder, indicating that systematic assessment is warranted. Half of higher-risk gambling FPPs reported pre-admission GP, whereas most had never sought help. Therefore, early screening could potentially benefit a substantial proportion of patients immediately. Our interviews with staff (11) reveal how GP among patients can be perceived as both difficult to assess and address, due in part to limited knowledge and the absence of organizational guidelines. Within the limits of the external validity of our GP prevalence estimates, this marks a gap in current care. It is essential that staff possess foundational knowledge of behavioral indicators of GP and have access to clear organizational guidelines to support clinical practice. Due to the complexity of the structures and content of forensic psychiatric practice, any initiatives, such as organizational guidelines, may be most effectively integrated through collaborative implementation studies involving researchers, patients, staff, and experts in GP.

### 4.2 Study limitations and strengths

The present study is the first of its kind and provides novel findings with important clinical implications. Nonetheless, several limitations should be noted. First, there is a risk of sampling bias. A total of 105 FPPs who were severely mentally ill or otherwise unable to complete the survey during the data collection period were excluded, and 56% of invited FPPs declined participation. Non-responder analysis was precluded due to GDPR constraints, and it remains unclear whether FPPs not included in the study were more or less susceptible to GP. However, our sample characteristics align with epidemiological overviews of FPPs, being predominantly young-to-middle-aged men diagnosed with psychotic disorders (5), suggesting some generalizability to forensic psychiatric populations outside the present study. Second, the results rely on self-reports and are therefore subject to biases such as social desirability. This may explain why respondents who required assistance with questionnaire completion reported lower rates of past-year gambling. Alternatively, a confounder may underlie both the need for assistance and a lower propensity to engage in gambling. Although the extent to which FPPs are generally prone to self-report bias remains uncertain, consistent associations between GP, gender, socioeconomic factors, self-exclusion, and criminality in our data lend support to the validity of the findings. Importantly, the self-report format also has practical value, as the results reflect patients’ subjective experiences and perceptions regarding their gambling behavior and GP. This is an essential dimension that clinical practice should also consider (50). Third, the generalizability of our findings may be limited due to cross-national differences in legislation governing forensic psychiatric practice (e.g., internet access) and gambling regulation (e.g., extent of legalization), highlighting the need for similar studies in other countries. Overall, our results can be interpreted in relation to the broader body of research on gambling problem prevalence, which also relies heavily on self-report measures such as PGSI (22), including the national survey from which our normative sample was drawn (30). Finally, given the cross-sectional design, the large number of comparisons, and the wide confidence intervals surrounding several effect estimates, all associations should be interpreted with caution. Future studies should strive to replicate our findings, preferably using longitudinal designs that incorporate diagnostic interviews alongside self-reported GP to support causal inference and to evaluate the validity of patients’ self-perceptions.

On balance, our study contributes important insights to consider in recovery-oriented approaches within forensic psychiatric practice, and sheds light on a clinical concern whose resolution requires careful integration within established governance frameworks.

## 5 Conclusion

This study provides the first systematic evidence that GP are highly prevalent among FPPs. Approximately 12% met criteria for problem gambling, indicative of gambling disorder. Rates markedly exceeded those in a gender- and age-matched normative sample. The findings may have implications for prevention and assessment efforts, and future studies should examine whether GP interfere with both FPP recovery and rehabilitation and increase recidivism risk.

## Data Availability

The raw data supporting the conclusions of this article will be made available by the authors, without undue reservation.
