# Supplementary material for: Prevalence and correlates of gambling problems among forensic psychiatric patients: evidence of elevated rates from a multi-site study
Source: Front Psychiatry. 2026 Oct 20;17:1931287. doi: 10.3389/fpsyt.2026.1931287 (PMC13727565; doi:10.3389/fpsyt.2026.1931287)
Supplement: Supplementary file 1 [file Table1.docx]

| **Supplementary Table 1**. Comparison of past-year non-gambling and gambling forensic psychiatric patients | | | |
| --- | --- | --- | --- |
|  | **Non-gambling FPPs**^a^ | **Gambling FPPs**^b^ | **Test p-value**^c^ |
| N | 116 | 98 |  |
| **Sociodemographics** | Mean (SD) / n (%) | |  |
| Age | 40.28 (12.21) | 35.76 (10.11) | **0.004** |
| Female^d^ | 26 (22%) | 17 (18%) | 0.376 |
| Country of birth: Denmark | 88 (77%) | 76 (78%) | 0.859 |
| Marital status: In relationship | 14 (13%) | 9 (10%) | 0.563 |
| Educational attainment > Primary school | 49 (42%) | 30 (31%) | 0.089 |
|  |  |  |  |
| **Financial characteristics** |  |  |  |
| Monthly income > 10.000 DKK | 80 (70%) | 74 (78%) | 0.207 |
| Monthly monetary savings (yes) | 52 (55%) | 52 (59%) | 0.552 |
| Consumer loans currently (yes) | 20 (19%) | 27 (30%) | 0.053 |
|  |  |  |  |
| **Clinical characteristics** |  |  |  |
| Hospitalization status: Inpatient | 43 (37%) | 44 (45%) | 0.245 |
| Length-of-stay |  |  |  |
| <1 years | 10 (14%) | 13 (20%) | 0.536 |
| 1-2 years | 6 (8.5%) | 10 (15%) |  |
| 2-3 years | 13 (18%) | 13 (20%) |  |
| 3-4 years | 7 (9.9%) | 6 (9.2%) |  |
| 4-5 years | 8 (11%) | 7 (11%) |  |
| 5+ years^e^ | 27 (38%) | 16 (25%) |  |
| SUD diagnosis^f^ | 34 (39%) | 36 (43%) |  |
|  |  |  |  |
| **Gambling variables** |  |  |  |
| Pre-admission gambling problems | 8 (8.6%) | 26 (30%) | **<0.001** |
| Current use of national gambling exclusion | 6 (15%) | 21 (30%) | 0.083 |
| Abbreviations: DKK; Danish Currency (Danske Kroner), N; number of participants, SD; standard deviation, SUD; substance use disorders. ^a^FPPs reporting no past-year gambling. ^b^FPPs reporting past-year gambling. ^c^Independent t-tests for continuous variables, χ^2^ or Fisher’s exact test for categorical variables. ^d^Participants were presented with the option of "other gender", but none chose this. ^e^Due to a reorganization of regional databases in February 2019, cases with a duration exceeding five years could not be dated. ^f^Counting both primary and secondary diagnoses. All tests were based on people responding to the given set of questions and thus did not include differences in propensity to be missing. p-values < 0.05 are shown in bold. | | | |

***Supplementary Material***

**Prevalence and correlates of gambling problems among forensic psychiatric patients: Evidence of elevated rates from a multi-site study**
